# Breed-specific responses to coccidiosis in chickens: identification of intestinal bacteria linked to disease resistance

**DOI:** 10.1186/s40104-025-01202-z

**Published:** 2025-05-08

**Authors:** Chace Broadwater, Jiaqing Guo, Jing Liu, Isabel Tobin, Melanie A. Whitmore, Michael G. Kaiser, Susan J. Lamont, Guolong Zhang

**Affiliations:** 1 Department of Animal and Food Sciences, Oklahoma State University, Stillwater, OK 74078 USA; 2 Department of Animal Science, Iowa State University, Ames, IA 50011 USA

**Keywords:** Coccidiosis, *Eimeria*, *Enterococcus*, Fayoumi, Lactic acid bacteria, *Lactobacillus*, Microbiota, Probiotics, *Staphylococcus*, *Weissella*

## Abstract

**Background:**

Coccidiosis, caused by *Eimeria* parasites, is a major enteric disease in poultry, significantly impacting animal health, production performance, and welfare. This disease imposes a substantial economic burden, costing the global poultry industry up to $13 billion annually. However, effective mitigation strategies for coccidiosis remain elusive. While different chicken breeds exhibit varying resistance to coccidiosis, no commensal bacteria have been directly linked to this resistance.

**Methods:**

To assess relative resistance of different breeds to coccidiosis, 10-day-old Fayoumi M5.1, Leghorn Ghs6, and Cobb chickens were challenged with 50,000 sporulated *Eimeria maxima* oocysts or mock-infected. Body weight changes, small intestinal lesions, and fecal oocyst shedding were evaluated on d 17. Ileal and cecal digesta were collected from individual animals on d 17 and subjected to microbiome analysis using 16S rRNA gene sequencing.

**Results:**

Fayoumi M5.1 chickens showed the lowest growth retardation, intestinal lesion score, fecal oocyst shedding, and pathobiont proliferation compared to Ghs6 and Cobb chickens. The intestinal microbiota of M5.1 chickens also differed markedly from the other two breeds under both healthy and coccidiosis conditions. Notably, group A *Lactobacillus* and *Ligilactobacillus salivarius* were the least prevalent in both the ileum and cecum of healthy M5.1 chickens, but became highly enriched and comparable to Ghs6 and Cobb chickens in response to coccidiosis. Conversely, *Weissella, Staphylococcus gallinarum*, and *Enterococcus durans/hirae* were more abundant in the ileum of healthy M5.1 chickens than in the other two breeds. Despite being reduced by *Eimeria*, these bacteria retained higher abundance in M5.1 chickens compared to the other breeds.

**Conclusions:**

Fayoumi M5.1 chickens exhibit greater resistance to coccidiosis than Leghorn Ghs6 layers and Cobb broilers. Several commensal bacteria, including group A *Lactobacillus*, *L. salivarius*, *Weissella*, *S. gallinarum*, and *E. durans/hirae*, are differentially enriched in Fayoumi M5.1 chickens with strong correlation with coccidiosis resistance. These bacteria hold potential as probiotics for coccidiosis mitigation.

**Supplementary Information:**

The online version contains supplementary material available at 10.1186/s40104-025-01202-z.

## Introduction

Coccidiosis, caused by the obligate intracellular parasite *Eimeria*, has been a significant poultry disease for decades [1, 2]. This disease is estimated to cause annual losses of up to $13 billion globally in broiler production [3]. Therefore, it is crucial to explore more effective strategies to combat this economically important disease. Among the seven species of *Eimeria* that commonly infect chickens, *E. maxima*, *E. tenella*, and *E. acervulina* are notable for their distinct morphology, tissue specificity, and pathogenicity [4]. For instance, *E. maxima* is moderately pathogenic and primarily targets the jejunum and ileum of the small intestine [4, 5].

The life cycle of *Eimeria* is well-documented and involves several stages [4, 5]. Upon ingestion, sporozoites are released from sporulated oocysts and invade intestinal epithelial cells. This invasion leads to cellular damage through multiple rounds of asexual and sexual reproduction, culminating in cell rupture. The life cycle is completed within 6–7 d, resulting in the development and excretion of new oocysts in the feces. This process compromises the intestinal barrier, impairs nutrient absorption, reduces growth performance, and can even lead to death in affected animals [4, 5].

The intestinal microbiome plays a vital role in protecting the host from invading pathogens. Commensal bacteria contribute to disease resistance by modulating the immune system, secreting antimicrobial compounds, supporting intestinal mucosal integrity, and competing for nutrients [6, 7]. Coccidiosis is known to disrupt the intestinal microbiota, leading to dysbiosis, proliferation of pathobionts, and impaired disease resistance [8–10]. Microbiome manipulation may offer potential for coccidiosis mitigation. Consistently, several probiotic products have been shown to alleviate coccidiosis in chickens [11, 12].

Chicken breeds exhibit varying levels of disease resistance. The Fayoumi line M5.1, derived from the Egyptian Fayoumi breed, is highly inbred and considered more resistant to many diseases than Leghorn chickens [13–15]. In contrast, the Leghorn line Ghs6 is a highly inbred line derived from the European Leghorn breed, which served as a foundation stock for many commercial layers [16]. Cobb broilers, a major commercial meat-type chicken breed, are known for their relative susceptibility to coccidiosis and necrotic enteritis [17]. This study aims to compare the resistance of these three chicken breeds to coccidiosis and to further investigate their intestinal microbiome differences. We hypothesize that breed-specific intestinal microbiome compositions contribute to coccidiosis resistance. Identifying commensal bacteria that are differentially enriched in disease-resistant breeds may offer potential for developing probiotics to combat coccidiosis.

## Methods

### Chicken coccidiosis model

Newly hatched Fayoumi M5.1 and Leghorn Ghs6 straight-run chicks were obtained from Iowa State University (Ames, IA, USA), while male Cobb 500 by-product breeder chicks were sourced from the Cobb–Vantress Hatchery (Siloam Springs, AR, USA). All chicks were unvaccinated, arrived on the same day, and were housed in a single environmentally controlled room in the Animal Nutrition Physiology Center at Oklahoma State University under standard management practices recommended by Cobb-Vantress. Upon arrival, chicks were tagged with wing bands and distributed into 0.91 m × 0.91 m floor pens with 10–15 birds/pen and fresh pinewood shavings. Tap water and a standard corn and soybean-based starter mash diet containing 21.5% crude protein were provided ad libitum throughout the trial. The diet was formulated to meet or exceed all nutrient requirements of broilers according to the 1994 recommendations of National Research Council [18]. Plastic sheeting was used to separate breeds and treatments, and extreme cautions were exercised to minimize cross-contamination during animal care and sampling.

Coccidiosis was induced on d 10 with 10–15 chicks/breed after overnight fasting. Animals were weighed individually and orally inoculated with 50,000 sporulated oocysts of *Eimeria maxima* strain M6 (kindly provided by Dr. John Barta at the University of Guelph, Canada) suspended in 1 mL of sterile saline, while mock-infected groups were inoculated with 1 mL saline as previously described [19, 20]. Chicks were closely monitored twice daily for signs of clinical symptoms or behavioral changes.

At 7 d post-infection (dpi), animals were weighed individually, and 7–10 fresh fecal droppings were collected from each group before animals were sacrificed via CO_2_ asphyxiation. The entire jejunum and ileum were examined, and lesion scores were assigned on a scale of 0–4 as described [21]. Additionally, approximately 0.5 g of proximal ileal digesta and approximately 0.2 g of the cecal digesta were collected from each animal. Samples were flash-frozen in liquid nitrogen and stored at −80 °C until further analysis. The animal experiment was performed in accordance with the Guide for the Care and Use of Agricultural Animals in Research and Teaching, 4^th^ edition [22]. All animal procedures were approved by the Institutional Animal Care and Use Committee of Oklahoma State University under protocol number AG-23-35.

### *Eimeria* oocyst counting

Fresh fecal samples were collected at 7 dpi to quantify *Eimeria* shedding using a salt flotation technique as described [23]. Briefly, each sample was weighed and resuspended in nine volumes of water before being filtered through four layers of sterile gauze pads. The oocysts were floated on a 35% sodium nitrate solution and counted on a McMaster chamber slide under 10 × magnification to determine oocysts per gram of the feces (OPG).

### Bacterial DNA isolation and 16S rRNA gene sequencing

Microbial genomic DNA was extracted from the ileal and cecal digesta using Fecal DNA MicroPrep and MiniPrep kits (Zymo Research, Irvine, CA, USA), respectively. DNA concentration and quality were assessed using a Nanodrop One Spectrophotometer (Thermo Fisher Scientific, Waltham, MA, USA). Samples were submitted for PE250 deep sequencing of the V3–V4 region of the 16S rRNA gene. Sequencing and library preparation were performed commercially by Novogene (Beijing, China) on an Illumina NovaSeq 6000 using primers (341 F: 5′-CCTAYGGGRBGCASCAG-3′ and 806R: 5′-GGACTACNNGGGTATCTAAT-3′).

### Bioinformatic analysis

The raw sequencing reads of the 16S rRNA gene were processed using QIIME 2 v2023.9 [24] as we described [20, 25]. After adapter and primer removal, paired read joining, and quality control, short reads were trimmed to 402 nucleotides and denoised using Deblur [26]. Bacterial amplicon sequence variants (ASVs) were classified against the Ribosomal Database Project (RDP) database using Bayesian classifier 2.14 (August 2023) [27] with 97% confidence. The identities of the top 100 ASVs were further confirmed and reclassified, if necessary, against the EzBioCloud 16S database (v2023.08.23) [28] at 97% identity. Further downstream analysis and visualization were conducted in R (v4.4.1) using ASVs that appeared in more than 5% of samples. The ‘phyloseq’ R package v1.48.0 [29] was used to calculate the number of ASVs, Pielou’s evenness, and Shannon index to indicate the richness, evenness, and overall α-diversity of the samples, respectively. The β-diversity was calculated using weighted and unweighted UniFrac distances. Raw read counts were used for differential abundance analysis using ANCOM-BC, v2.6.0 [30].

### Statistical analysis

Statistical analyses were performed using GraphPad Prism version 10.0.0 (Boston, Massachusetts, USA) or R (v4.4.1) using parametric or non-parametric methods, depending on the normality of the data determined by the Shapiro-Wilk test and the homogeneity of variance across groups determined by the Levene’s test. Statistical significance of weight gain was determined using an unpaired Student’s *t*-test between the mock-infected group and their corresponding *Eimeria* challenge group for each breed. OPG significance was determined using one-way analysis of variance, followed by Tukey’s test for pairwise comparisons. Intestinal lesion scores were analyzed using the nonparametric Kruskal-Wallis test, followed by Dunn’s test for pairwise comparisons. The α-diversity indices were analyzed using the Mann-Whitney test between the mock and infected groups within each breed, while β-diversity was compared among groups using permutational multivariate analysis of variance (PERMANOVA) with 999 permutations using ‘vegan’ package v2.6-8 [31]. *P* < 0.05 was considered statistically significant.

## Results

### Changes in body weight, intestinal lesions, and fecal shedding of oocysts in response to coccidiosis

**Fig. 1:**
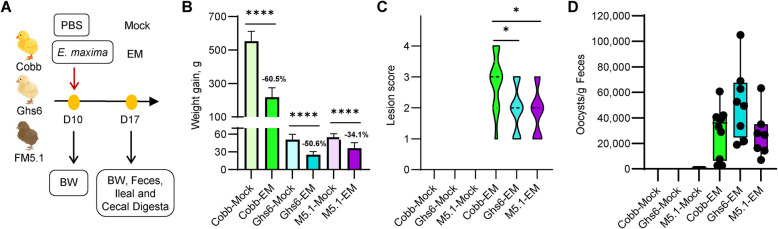
Experimental design, growth performance, intestinal lesion scores, and oocyst shedding among three chicken breeds in response to coccidiosis. **A** Cobb, Leghorn Ghs6, and Fayoumi M5.1 chickens (*n* = 10–15) were challenged with sporulated *E. maxima* (EM) or mock-infected on d 10. Body weight (BW) was recorded for individual birds on d 10 and 17. Small intestinal lesion was scored for each bird, and fecal samples were collected on d 17. **B** Average weight gain of individual birds in each group between d 10 and 17. Each EM group was compared to the respective mock group within each breed using Student’s *t*-test. Error bars indicate the standard deviation. The percentage decrease in weight gain is indicated above each challenge group. **C** Violin plot of intestinal lesion score of each group, with significance determined by the nonparametric Kruskal-Wallis test, followed by Dunn’s pairwise comparisons. No lesions were observed in mock-infected groups. **D** Box plot of oocysts per gram of feces values from individual birds. Levels of statistical significance are indicated according to one-way ANOVA. No oocysts were observed in the feces of mock-infected birds. ^*^P < 0.05, ^****^P < 0.0001

To directly compare relative resistance to coccidiosis, newly hatched Cobb, Leghorn Ghs6, and Fayoumi M5.1 lines of chickens were subjected to identical management and challenged separately with 50,000 sporulated *E. maxima* oocysts on d 10 (Fig. 1A). Although there was no coccidiosis-related lethality in any of the groups throughout the duration of this study, clinical symptoms, including lethargy, general malaise, ruffled feathers, and bloody diarrhea, were observed in great than 80% of infected Cobb chickens, but in less than 10% of Ghs6 and M5.1 chickens. Consistently, *E. maxima* caused a significant decrease in weight gain between d 10 and 17 in all three breeds (*P* < 0.0001) when compared to their mock-infected counterparts (Fig. 1B). However, the rate of growth retardation differed for each breed. Relative to their mock-infected counterparts, *E. maxima-*challenged Cobb, Ghs6, and M5.1 chickens demonstrated a 60.5%, 50.6%, and 34.1% decrease in weight gain (*P* < 0.0001), respectively (Fig. 1B).

Intestinal lesion scores are categorized on a scale of 0 to 4, with 0 meaning no lesions and 4 indicative of severe intestinal damage, as previously described [21]. While mock-infected groups showed no sign of disease, each *Eimeria* challenge group presented visible intestinal damage. Cobb chickens developed the most severe lesions, with an average score of 2.9, while Ghs6 and M5.1 chickens had a significantly reduced lesion score of 1.9 (*P* < 0.05; Fig. 1C). The median OPG values observed at 7 dpi were 3.6 × 10^4^, 5.2 × 10^4^, and 2.7 × 10^4^ for Cobb, Ghs6, and M5.1 chickens, respectively (Fig. 1D). Ghs6 chickens tended to shed more oocysts in the feces than M5.1 chickens (*P* = 0.07). Although statistically insignificant, Cobb chickens shed more oocysts than M5.1 chickens (Fig. 1D). Given that Cobb chicks are 4.5 times heavier than M5.1 chickens (614.2 g vs. 135.9 g) at 7 dpi and that Cobb chickens had significantly increased watery fecal content due to severe diarrhea, 5–10 times more oocysts are likely to persist in the intestinal tract of a Cobb chicken than that of a Ghs6 or M5.1 chicken. Collectively, these results suggested that Fayoumi M5.1 chickens are more resistant to *E. maxima* infection than Leghorn Ghs6 chickens, while Cobb broilers are the most susceptible.

### Coccidiosis-induced alterations of the ileal microbiome

To investigate the differential response of the intestinal microbiome to coccidiosis among three chicken breeds, bacterial DNA was isolated from both the ileal and cecal digesta of each breed following infection. A total of 157 samples yielded 10,096,962 high-quality sequences with an average of 64,312 ± 4,357 sequences per sample. After denoising and filtering out ASVs present in less than 5% of samples, 450 ASVs were retained for both the ileum and cecum.

**Fig. 2:**
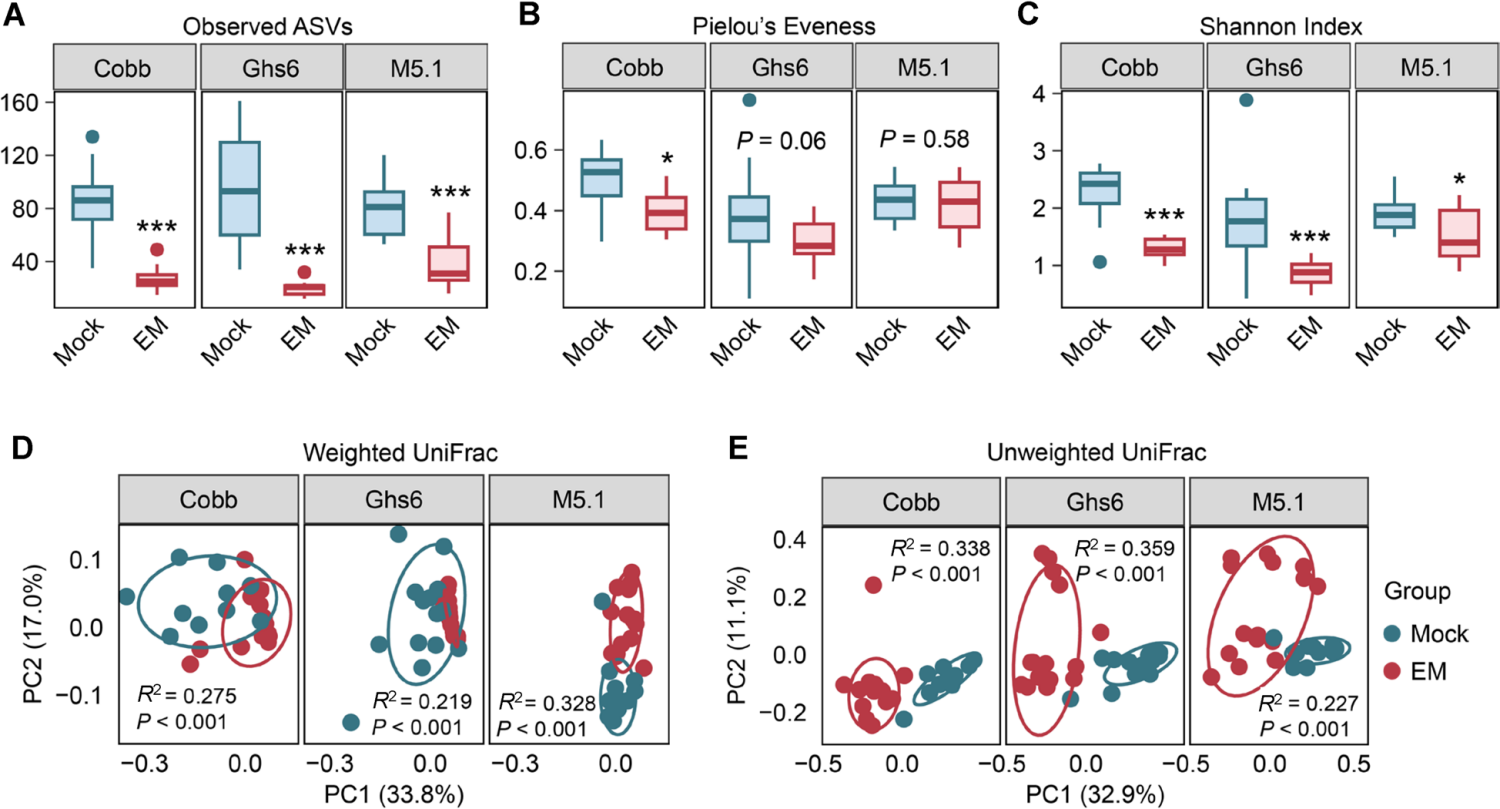
Alterations in α- and β-diversities of the ileal microbiome among three chicken breeds in response to coccidiosis. Cobb, Leghorn Ghs6, and Fayoumi M5.1 chickens were challenged with sporulated *E. maxima* (EM) or mock-infected on d 10. Ileal digesta samples (*n* = 10–15) were collected on d 17 and subjected to DNA isolation and 16S rRNA gene sequencing. Box plots of observed amplicon sequence variants (ASVs) (**A**), Pielou’s Evenness index (**B**), and Shannon Index (**C**) were calculated for each group. Statistical analysis was performed using the nonparametric Mann-Whitney U test for each breed. ^*^P < 0.05, ^***^P < 0.001. Principal coordinate analysis (PCoA) plots of weighted UniFrac (**D**) and unweighted UniFrac distances (**E**) are shown for each breed with or without EM challenge. Statistical significance was determined using PERMANOVA with 999 permutations and is indicated in each panel

In the ileum, the number of observed ASVs, a measure of bacterial richness, significantly decreased (*P* < 0.001) in *Eimeria*-challenged chickens for each breed (Fig. 2A). Pielou’s Evenness, a measure of species evenness, showed a significant reduction in infected Cobb chickens (*P* < 0.05) and tended to decrease in Ghs6 chickens (*P* = 0.06), while no significant change was observed in infected M5.1 chickens (*P* = 0.58) (Fig. 2B). The Shannon index, an indicator of overall α-diversity, significantly declined (*P* < 0.05) in each *Eimeria*-challenged group compared to healthy counterparts, with the reduction being less substantial in M5.1 chickens than the other two breeds (Fig. 2C).

Weighted and unweighted UniFrac distances, which measure the uniqueness of each microbiome sample based on the phylogenetic relationships of individual bacteria, showed significant differences (Fig. 2D and E). These differences were further confirmed in most pairwise comparisons (Table S1), suggesting that the ileal microbiomes differ significantly among the breeds and that coccidiosis drastically alters the ileal microbiome.

**Fig. 3:**
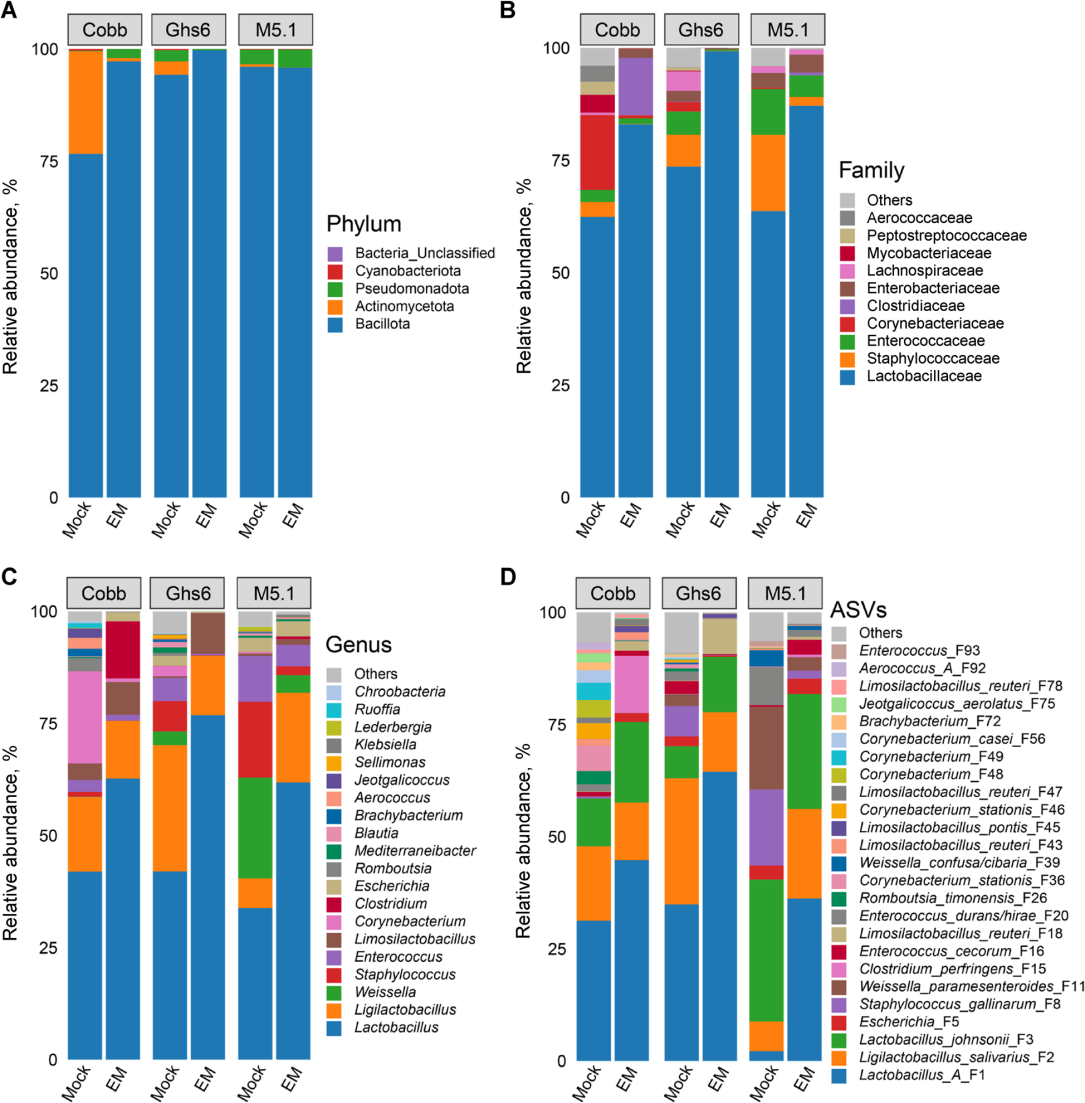
Alterations in relative abundances (%) of the ileal microbiome among three chicken breeds in response to coccidiosis. Cobb, Leghorn Ghs6, and Fayoumi M5.1 chickens were challenged with sporulated *E. maxima* (EM) or mock-infected on d 10. Ileal digesta samples (*n* = 10–15) were collected on d 17 and subjected to 16S rRNA gene sequencing. The compositional changes in the ileal microbiome are shown for different groups of chickens at the phylum (**A**), family (**B**), genus (**C**), and amplicon sequence variant (ASV) (**D**) levels

Compositionally, the ileal microbiome of heathy, d-17 Cobb chickens is different from those of Ghs6 and M5.1 chickens at the phylum level. The most abundant ileal bacterial phylum Bacillota, also known as Firmicutes, accounted for approximately 95% in healthy Ghs6 and M5.1 chicks, but only amounted to 77% in healthy Cobb chickens (Fig. 3A and Table S2). Conversely, the ileum of Cobb chickens harbored 23% of Actinomycetota, which was largely absent in the other two breeds. Actinomycetota were substantially reduced in all three breeds in response to *Eimeria* infection (Fig. 3A and Table S2).

Among the top four bacterial families in the ileum, Lactobacillaceae was dominant and comparable among the three breeds, and was further enriched following infection (Fig. 3B). On the other hand, M5.1 chickens harbored more abundant Staphylococcaceae and Enterococcaceae than Ghs6 chickens, with Cobb chickens harboring the least. However, both bacterial families were greatly diminished in all three breeds in response to *Eimeria* infection (Fig. 3B). Corynebacteriaceae*,* belonging to Actinomycetota, accounted for 17% of total bacteria in the ileum of Cobb chickens but was largely absent in the other two breeds. Corynebacteriaceae was also greatly diminished by *Eimeria* in Cobb chickens (Fig. 3B and Table S2).

At the genus level, *Lactobacillus* was the most prevalent and comparable in all three breeds and further increased following *Eimeria* infection, while no consistent trend was observed for *Ligilactobacillus* or *Limosilactobacillus* (Fig. 3C and Table S2). Conversely, *Weissella*, another genus of lactic acid bacteria, was uniquely abundant in M5.1 chickens, amounting to 22.5%, while largely absent in the other two breeds. *Weissella* was also significantly diminished in response to coccidiosis across all three breeds (Table S2).

At the ASV level, group A *Lactobacillus* (F1), which consists of highly related species such as *L. acidophilus*, *L. crispatus*, *L. gallinarum,* and *L. kitasatonis* that cannot be distinguished from sequencing the V3–V4 region of the bacterial 16S rRNA gene, were enriched in response to *E. maxima* in all three chicken breeds, while group B *Lactobacillus* (*L. johnsonii* F3) increased only in Cobb and Ghs6 chickens (Fig. 3D and Table S2). Notably, while the total amount of *Lactobacillus* was comparable among the three breeds, *L. johnsonii* (F3) was more prevalent and group A *Lactobacillus* (F1) was much less abundant in M5.1 chickens than in the other two breeds. *Ligilactobacillus salivarius* (F2) was not significantly altered by *Eimeria* in Cobb and Ghs6 chickens but experienced significant enrichment in M5.1 chickens. Conversely, *Weissella paramesenteroides* (F11) was greatly reduced in all three breeds during coccidiosis (Fig. 3D and Table S2).

**Fig. 4:**
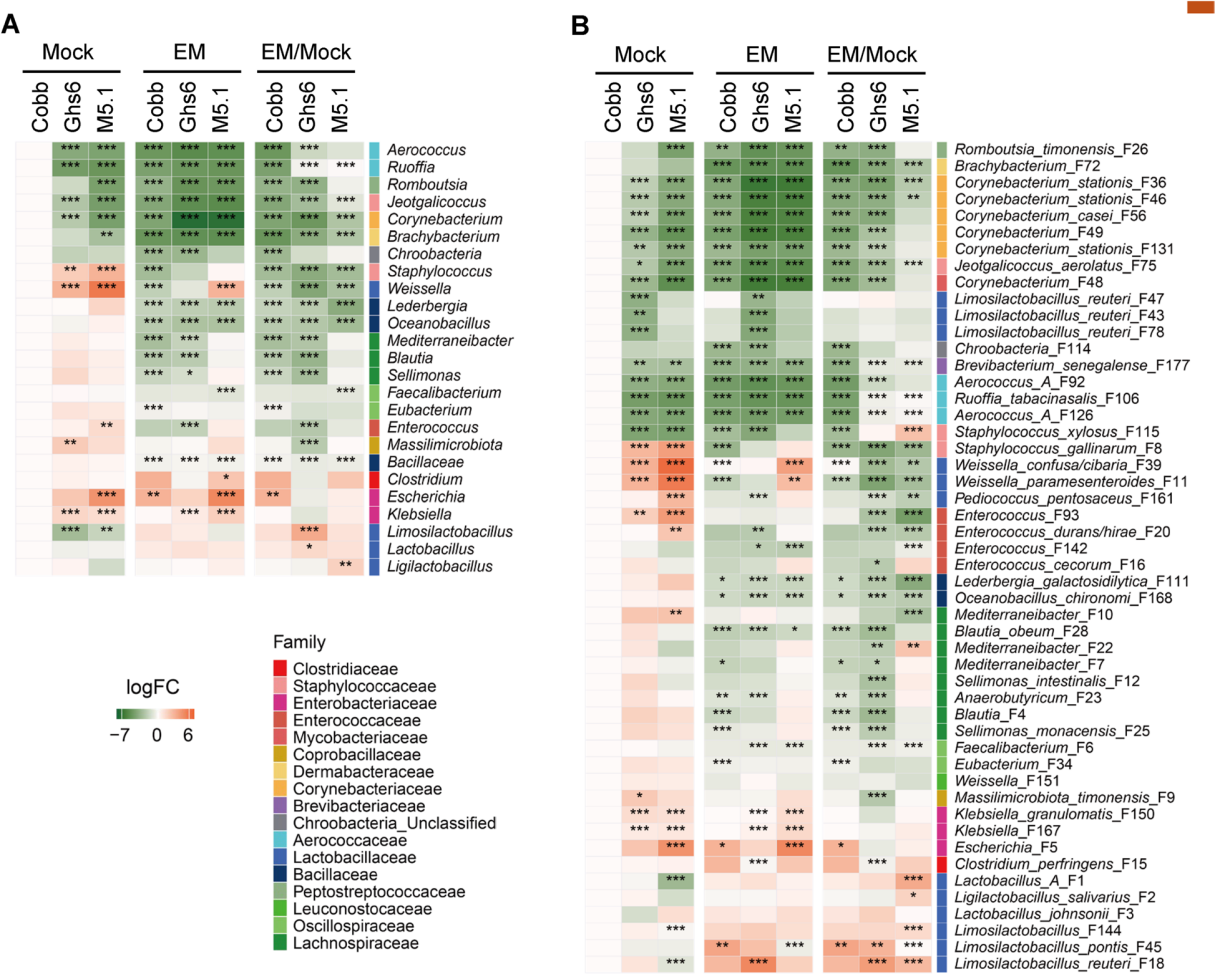
Differential abundance of the ileal microbiome among three chicken breeds in response to coccidiosis. Cobb, Leghorn Ghs6, and Fayoumi M5.1 chickens were challenged with sporulated *E. maxima* (EM) or mock-infected on d 10. Ileal digesta samples (*n* = 10–15) were collected on d 17 and subjected to 16S rRNA gene sequencing. Differential abundance analysis of the ileal microbiome at the genus (**A**) and amplicon sequencing variant (ASV) (**B**) levels was performed using ANCOM–BC [30]. The left two panels in each heatmap depict log_2_ transformations of the fold changes of differentially enriched bacteria in the ileum of mock and EM-infected chickens, relative to mock-infected Cobb chickens. The right panel in each heatmap illustrates log_2_ transformations of fold differences of individual bacteria between EM- and mock-infected chickens in each breed. ASVs are further color-coded by family. ^*^P < 0.05, ^**^P < 0.01, and ^***^P < 0.001

ANCOM-BC analysis [30] was further applied to reveal differentially abundant bacteria at both the genus and ASV levels in response to coccidiosis among the three breeds of chickens. While most bacteria were reduced by the *Eimeria* challenge, group A *Lactobacillus* (F1) and *L. salivarius* (F2) showed significant blooms in M5.1 chickens, whereas the most susceptible Cobb chickens showed an enrichment of *Escherichia* (F5) (Fig. 4A and B). Additionally, three *Limosilactobacillus* species (F18, F45, and F144) were uniformly enriched in all three breeds of chickens in response to coccidiosis (Fig. 4A and B).

### Coccidiosis-induced alterations of the cecal microbiome

**Fig. 5:**
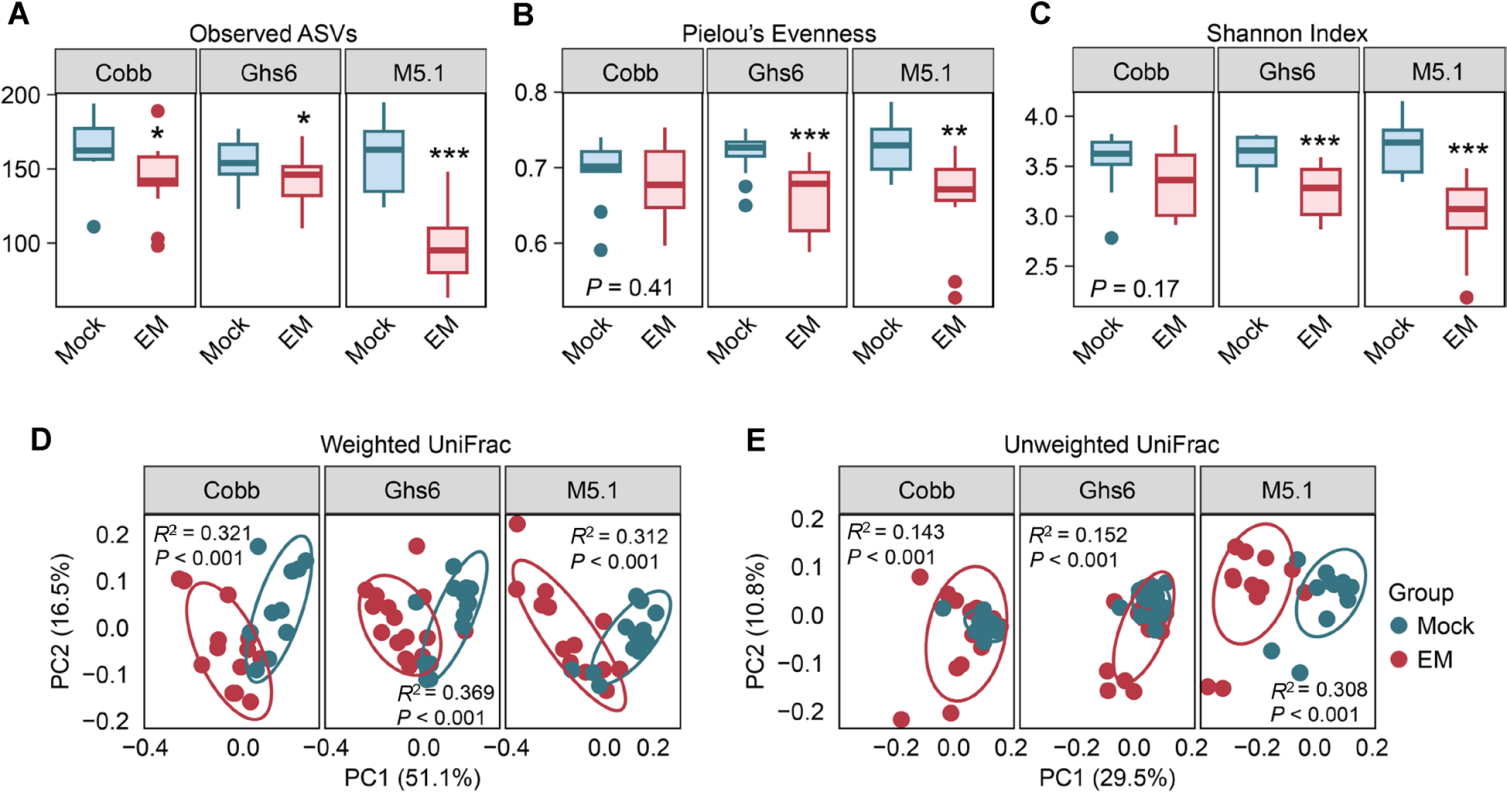
Alterations in α- and β-diversities of the cecal microbiome among three chicken breeds in response to coccidiosis. Cobb, Leghorn Ghs6, and Fayoumi M5.1 chickens were challenged with sporulated *E. maxima* (EM) or mock-infected on d 10. Cecal digesta samples (*n* = 10–15) were collected on d 17 and subjected to DNA isolation and 16S rRNA gene sequencing. Box plots of observed amplicon sequence variants (ASVs) (**A**), Pielou’s Evenness index (**B**), and Shannon Index (**C**) were calculated for each group. Statistical analysis was performed using the nonparametric Mann-Whitney U test for each breed. ^*^P < 0.05, ^**^P < 0.01 ^***^P < 0.001. Principal coordinate analysis (PCoA) plots of weighted UniFrac (**D**) and unweighted UniFrac distances (**E**) are shown for each breed with or without EM challenge. Statistical significance was determined using PERMANOVA with 999 permutations and is indicated in each panel

Similar to the ileum, the number of observed ASVs significantly decreased in the cecum across all three chicken breeds following *Eimeria* challenge (Fig. 5A). Both M5.1 and Ghs6 chickens showed a significant reduction in Pielou’s evenness, while Cobb chickens demonstrated no significant changes upon infection (*P* = 0.41) (Fig. 5B). The Shannon index of the cecal microbiome was unaffected by *Eimeria* challenge in Cobb chickens (*P* = 0.17), whereas M5.1 and Ghs6 chickens experienced significant reductions (Fig. 5C). Both weighted and unweighted UniFrac distances were significantly different among the three chicken breeds under both healthy conditions and coccidiosis (Fig. 5D and E). Pairwise comparisons further confirmed that most pairs were significantly different in both weighted and unweighted UniFrac distances (Table S3).

**Fig. 6:**
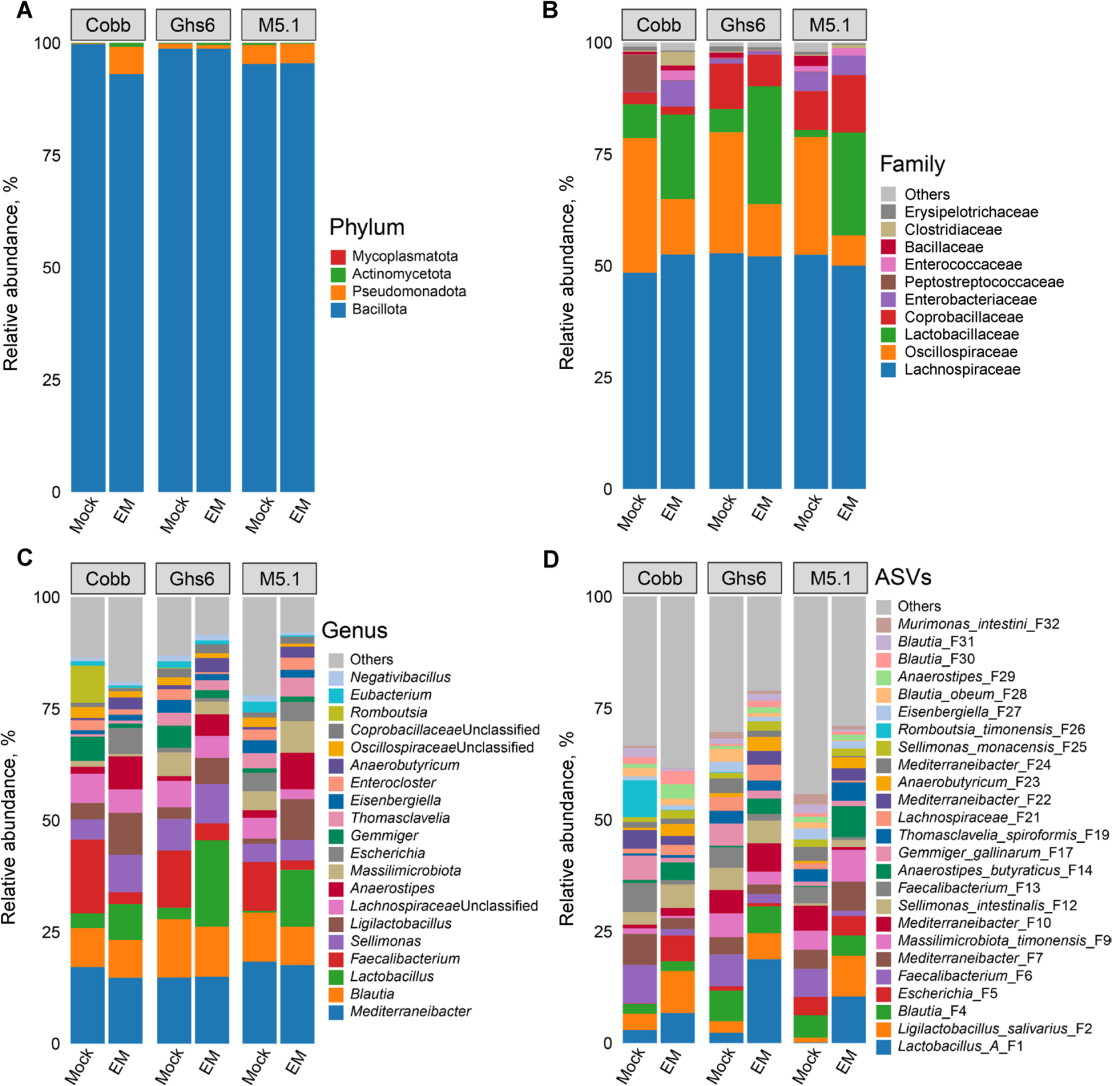
Alterations in relative abundances (%) of the cecal microbiome among three chicken breeds in response to coccidiosis. Cobb, Leghorn Ghs6, and Fayoumi M5.1 chickens were challenged with sporulated *E. maxima* (EM) or mock-infected on d 10. Cecal digesta samples (*n* = 10–15) were collected on d 17 and subjected to 16S rRNA gene sequencing. The compositional changes in the cecal microbiome are shown for different groups of chickens at the phylum (**A**), family (**B**), genus (**C**), and amplicon sequence variant (ASV) (**D**) levels

Compositionally, Bacillota dominated the cecal microbiome at the phylum level, accounting for > 95% of total bacteria in healthy chickens of all three breeds (Fig. 6A and Table S4). At the family level, the cecum was dominated by Lachnospiraceae, Oscillospiraceae, and Lactobacillaceae (Fig. 6B and Table S4). While Lachnospiraceae remained unchanged, Oscillospiraceae significantly diminished in all three breeds in response to coccidiosis (Fig. 6B and Table S4). Additionally, Lactobacillaceae were enriched in all three breeds of chickens following *Eimeria* challenge.

At the genus level, *Mediterraneibacter, Faecalibacterium,* and *Blautia* were the three most dominant genera in the cecum of healthy chickens (Fig. 6C). While *Mediterraneibacter* and *Blautia* showed no significant changes in response to *Eimeria* in any breed, *Faecalibacterium* was notably reduced, particularly in M5.1 chickens (Fig. 6C and Table S4). Concurrently, *Lactobacillus* significantly increased in both M5.1 and Ghs6 chickens during coccidiosis (*P* < 0.001), while *Ligilactobacillus* was significantly enriched only in M5.1 chickens (*P* < 0.001) (Fig. 6C and Table S4).

At the ASV level, short-chain fatty acid (SCFA)-producing bacteria, such as *Blautia* (F4, F28, F30, and F31), *Faecalibacterium* (F6, F13, and F44), and *Mediterraneibacter* (F7, F10, F22, and F24) were prevalent in the cecum of healthy chickens (Fig. 6D). Group A *Lactobacillus* (F1) and *L. salivarius* (F2) were relatively minor in healthy chickens, but markedly increased following *Eimeria* challenge (Fig. 6D and Table S4).

**Fig. 7:**
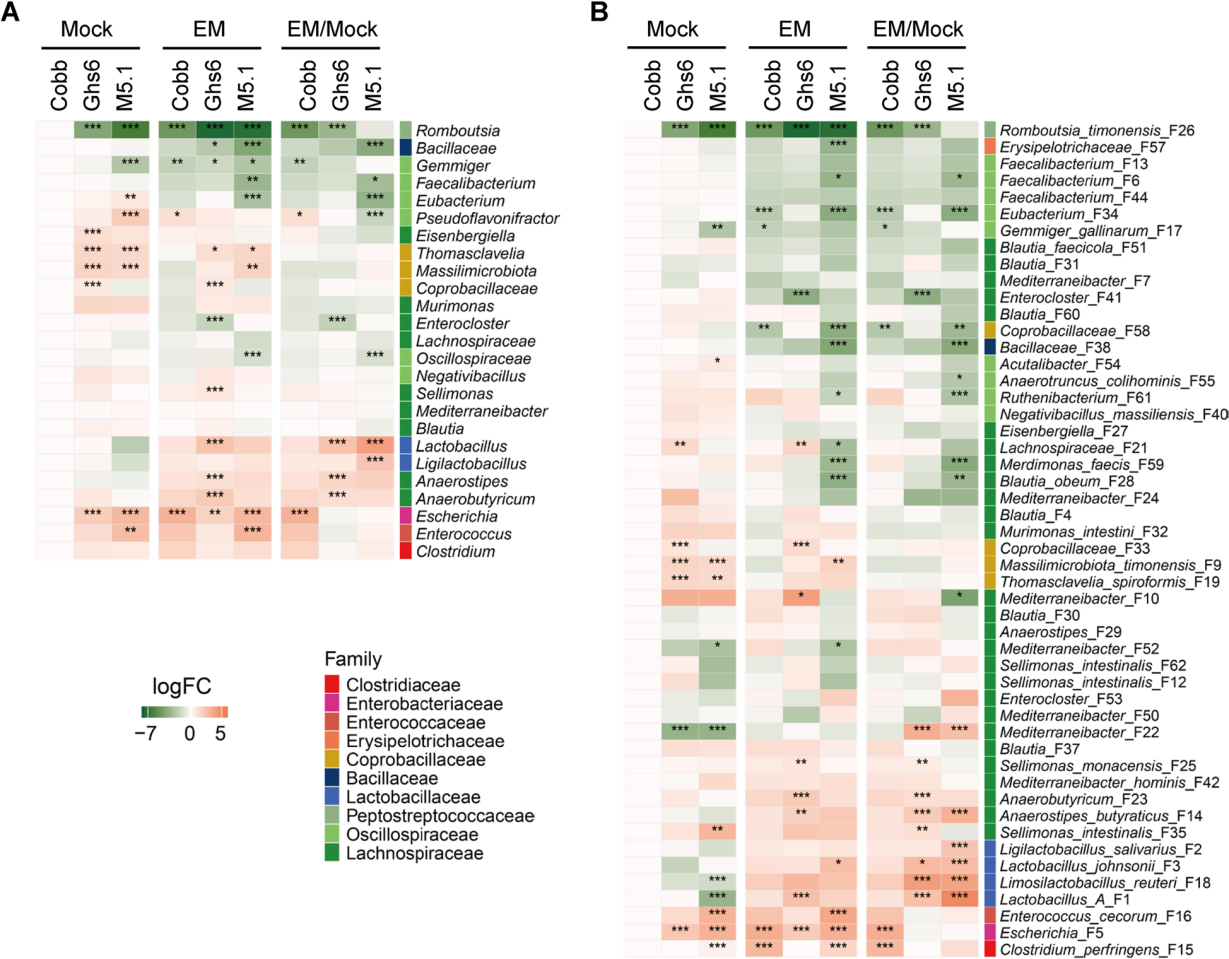
Differential abundance of the cecal microbiome among three chicken breeds in response to coccidiosis. Cobb, Leghorn Ghs6, and Fayoumi M5.1 chickens were challenged with sporulated *E. maxima* (EM) or mock-infected on d 10. Cecal digesta samples (*n* = 10–15) were collected on d 17 and subjected to 16S rRNA gene sequencing. Differential abundance analysis of the cecal microbiome at the genus (**A**) and amplicon sequencing variant (ASV) (**B**) levels was performed using ANCOM-BC [30]. The left two panels in each heatmap depict log_2_ transformations of the fold changes of differentially enriched bacteria in the cecum of mock and EM-infected chickens, relative to mock-infected Cobb chickens. The right panel in each heatmap illustrates log_2_ transformations of fold differences of individual bacteria between EM- and mock-infected chickens in each breed. ASVs are further color-coded by family. ^*^P < 0.05, ^**^P < 0.01, and ^***^P < 0.001

ANCOM-BC analysis [30] further revealed breed-specific responses to coccidiosis. Certain SCFA-producing bacteria, such as *Faecalibacterium* (F6 and F13) and *Blautia obeum* (F28), were reduced in the cecum of Cobb and Ghs6 chickens, with a more pronounced suppression in M5.1 chickens in response to *Eimeria* infection (Fig. 7A and B). At the same time, several species of lactic acid bacteria, such as group A *Lactobacillus* (F1), group B *Lactobacillus* (*L. johnsonii* F3), *L. salivarius* (F2), and *L. reuteri* (F18), were significantly enriched in M5.1 chickens but not in Cobb chickens. Most lactic acid bacteria, except for *L. salivarius* (F2), were also enriched in Ghs6 chickens following *Eimeria* challenge (Fig. 7B). Consequently, pathobionts such as *Escherichia* (F5) and *C. perfringens* (F15) significantly bloomed only in the susceptible Cobb chickens, but not in M5.1 or Ghs6 chickens (Fig. 7B).

## Discussion

### Fayoumi M5.1 chickens are highly resistant to *E. maxima*-induced coccidiosis

Coccidiosis is well-documented to cause severe growth retardation in chickens due to the damage it inflicts on intestinal epithelial cells, which impairs nutrient digestion and absorption [1–3]. This study aims to evaluate the resistance of three chicken breeds, including Cobb, Leghorn Ghs6, and Fayoumi M5.1, to *E. maxima*-induced coccidiosis. All three breeds exhibit a significant reduction in weight gain following *E. maxima* infection. However, the extent of this reduction varies among the breeds. Cobb chickens experience the greatest decline in weight gain, while M5.1 chickens show the least decline, with Ghs6 chickens displaying an intermediate response. Consistent with the weight gain changes, Cobb chickens have the highest average intestinal lesion scores, indicating severe intestinal damage. In contrast, two inbred lines, M5.1 and Ghs6, exhibit less severe lesions.

Additionally, the oocyst shedding patterns further support these findings. Ghs6 chickens shed more oocysts than M5.1 and Cobb chickens, and Cobb chickens also tend to shed more oocysts than M5.1 chickens, although this difference is statistically insignificant. The severe diarrhea and higher fluid content in the feces of Cobb chickens have likely contributed to an underestimation of their oocyst counts in the intestinal tract. Moreover, the higher body weight and greater intestinal capacity of *Eimeria*-challenged Cobb chickens compared to infected M5.1 chickens suggest that Cobb chickens harbor a much larger load of *Eimeria* following infection. This is further evidenced by more severe clinical symptoms observed in Cobb chickens. Additionally, Cobb chickens are nearly five times heavier than M5.1 or Ghs6 chickens at the time of challenge, but received the same amount of *Eimeria* as the other two breeds. If the *Eimeria* dose were adjusted by body weight, Cobb chickens would be expected to develop much more severe symptoms with more pronounced oocyst shedding in the feces.

Overall, our results clearly indicate that among the three breeds evaluated, Fayoumi M5.1 chickens demonstrate the highest resistance to *E. maxima*-induced coccidiosis, as evidenced by their improved growth performance, reduced intestinal lesions, minimal oocyst shedding, and mild clinical symptoms. On the other hand, Cobb chickens are the most susceptible, while Leghorn Ghs6 chickens exhibit moderate susceptibility. Robust coccidiosis resistance of the highly inbred Fayoumi M5.1 line is perhaps not surprising, given that this breed is known to resist various diseases, such as *Eimeria tenella*-induced coccidiosis, avian influenza, and Newcastle disease, compared to Leghorns [13–15, 32].

### Healthy intestinal microbiome differs substantially among three chicken breeds

The intestinal microbiome varies substantially among the three chicken breeds under healthy non-challenged conditions, particularly in the less complex ileal microbiome. Lactic acid bacteria, such as *Lactobacillus* and *Ligilactobacillus*, dominate the ileal microbiota, accounting for two-thirds of the total bacteria. These bacteria help reduce inflammation, strengthen the intestinal barrier, compete against pathogens, and support the healing of damaged gut lining [33, 34]. Group A *Lactobacillus* is dominant in the ileum of Cobb and Ghs6 chickens, accounting for approximately one-third of the total bacteria, while M5.1 chickens harbor approximately 15-fold less. Instead, group B *Lactobacillus* (*L. johnsonii*) is the most prevalent in M5.1 chickens, representing 32%, with only approximately 10% in the other two breeds. *L. salivarius* is also less abundant in M5.1 chickens than in the other two breeds. Additionally, *Weissella*, another genus of lactic acid bacteria, amounts to approximately one-quarter of the total bacterial population in M5.1 chickens but is minuscule in Ghs6 (3.1%) and especially Cobb chickens (0.07%). Consistently, *Weissella* species have been shown to be beneficial in alleviating gut inflammation and colitis [35–37].

Another unique feature of the M5.1 ileal microbiota is the substantially higher abundance of *Enterococcus durans* or *E. hirae* (8.4%) compared to Ghs6 or Cobb chickens (< 2%), while *E. cecorum* is comparable among all three breeds. This is perhaps not surprising, given that *E. durans* and *E. hirae* are known to be probiotic, while *E. cecorum* is often pathogenic [38]. Furthermore, M5.1 chickens harbor significantly higher levels of *Staphylococcus gallinarum* compared to Ghs6 and Cobb chickens. In contrast, Cobb chickens have a diverse population of *Corynebacterium* species, comprising approximately 20% of the ileal microbiome, whereas this genus is largely absent in the other two breeds. While *S. gallinarum* and *Corynebacterium* are generally considered innocuous, their specific physiological roles in the intestinal tract remain largely unknown. Although not typically pathogenic, both can opportunistically cause infections, particularly in hosts with weakened defenses [39–41].

Although complex, the cecal microbiome appears to be less diverse among the three chicken breeds. Major SCFA producers such as *Mediterraneibacter*, *Blautia*, *Faecalibacterium*, and *Sellimonas* were similar in the cecum of all three breeds. Similar to the ileum, group A *Lactobacillus* and *L. salivarius* are less abundant in M5.1 than in the other two breeds. The unique composition of the intestinal microbiome of M5.1 chickens could be partially responsible for their resistance to coccidiosis. It will be important to investigate the role of bacteria unique to M5.1 chickens, such as group A *Lactobacillus*, *L. salivarius*, *W. paramesenteroides*, *S. gallinarum*, and *E. durans/E. hirae*, in resistance to coccidiosis.

### Differential response of the intestinal microbiome to coccidiosis among three chicken breeds

Coccidiosis-induced dysbiosis is evident in both the ileum and cecum, aligning with previous studies. The infection by *Eimeria* significantly reduces bacterial richness and overall diversity across all three chicken breeds, reflecting a significant diminishment of various bacterial taxa. This infection also causes notable shifts in both ileal and cecal microbiota compositions, as demonstrated by changes in weighted and unweighted UniFrac distances. In the ileum, although healthy M5.1 chickens have the lowest levels of group A *Lactobacillus* and *L. salivarius*, these bacteria have significantly expanded following infection, reaching levels comparable to those found in Ghs6 and Cobb chickens.

Moreover, *Weissella*, *S. gallinarum*, and *E. durans/hirae* are highly enriched in the ileum of M5.1 chickens compared to the other two breeds. Despite a significant reduction in these bacteria due to *Eimeria* infection, they still constitute 1.5%–3% of the ileal microbiota in M5.1 chickens, whereas they are nearly abolished in Ghs6 and Cobb chickens. In the cecum, group A *Lactobacillus* and *L. salivarius* also increase in all three breeds in response to *Eimeria* infection, with the most pronounced increase observed in M5.1 chickens. Although *Eimeria* diminishes many SCFA-producing bacteria across all breeds, major producers like several *Faecalibacterium* and *Blautia* species show the greatest reduction in M5.1 chickens. Further research is needed to understand the role of these SCFA producers in disease resistance.

### Proliferation of pathobionts induced by coccidiosis

Coccidiosis is known to trigger dysbiosis, leading to the proliferation of opportunistic pathogens such as *Escherichia* and *C. perfringens* in poultry [8–10], which increases the risk of secondary bacterial infections. In fact, *Eimeria* is a significant predisposing factor for necrotic enteritis, and its infection can lead to the proliferation of *C. perfringens*, potentially resulting in necrotic enteritis [42]. In Cobb chickens, *Escherichia* and *C. perfringens* were enriched in both the cecum and ileum following *Eimeria* infection, consistent with previous findings [8, 20]. However, this trend was not observed in Ghs6 or M5.1 chickens. The absence of significant pathobiont proliferation in Fayoumi and Ghs6 chickens suggests that their microbiomes are more resistant to *Eimeria*-induced dysbiosis, and consequently, to coccidiosis. The blooming of pathobionts, which are often facultative or aerotolerant commensal bacteria, typically results from the reduction of obligate anaerobic commensals due to gut inflammation and the production of reactive oxygen and nitrogen species [43, 44].

### Limitations and future studies

In this study, we have revealed the unique intestinal microbiota composition of coccidiosis-resistant Fayoumi M5.1 chickens compared to Leghorn Ghs6 and Cobb chickens under healthy and coccidiosis conditions. The identification of differentially abundant bacteria in Fayoumi chickens presents promising opportunities for the development of novel probiotic candidates aimed at coccidiosis intervention. Given the broad-spectrum disease resistance exhibited by Fayoumi chickens, these bacteria may also hold potential for mitigating other diseases. [13–15, 32]. However, this study has several limitations.

Firstly, host genetics plays a major role in disease resistance. Although the intestinal microbiome is clearly different across breeds, this study did not provide direct evidence to conclude whether the microbiome contributes to coccidiosis resistance. It will be important to evaluate the resistance of chickens to coccidiosis after transferring the microbiome from disease-resistant M5.1 chickens to newly hatched susceptible Cobb chickens.

Secondly, the sex ratio of the birds varied across breeds, potentially influencing microbiome composition. Cobb chickens were all male, while the other two breeds were straight-run, with an unknown ratio of males and females. Although sex hormones can cause minor differences in microbiome composition [45], this study only involved chickens up to 17 days old, well before the 4–6 months when they are generally considered sextually mature. Therefore, sex should have had minimal or no influence on the outcome of this study.

Thirdly, the chicks were hatched at separate facilities, which could have impacted their initial microbial colonization [46]. Early microbial exposure is believed to play a significant role in microbiome development, and variations in initial exposure could contribute to the observed differences in microbiome composition [47]. However, recent studies suggest that the influence of the hatchery environment on microbiome development in poultry production is minimal [48].

Future studies should aim to include an equal number of male and female birds for each breed to control for sex-related microbiome differences. Additionally, it is crucial to investigate the role of bacteria uniquely present in coccidiosis-resistant Fayoumi M5.1 chickens, either individually or in combination, in mitigating coccidiosis. Group A *Lactobacillus* includes several closely related species such as *L. acidophilus*, *L. crispatus*, and *L. gallinarum*, which cannot be distinguished by sequencing the V3–V4 region of the bacterial 16S rRNA gene. Therefore, full-length 16S rRNA gene sequencing or shotgun metagenomic sequencing is necessary to accurately identify these species. It is important to determine which specific group A *Lactobacillus* species are present in M5.1 chickens and which species or strains undergo rapid expansion in response to coccidiosis.

## Conclusions

This study has confirmed that Fayoumi M5.1 chickens exhibit greater resistance to *E. maxima*-induced coccidiosis compared to Leghorn Ghs6 and Cobb chickens. Fayoumi chickens also harbor unique intestinal microbiota relative to the other two breeds. Specifically, Group A *Lactobacillus* and *L. salivarius* are significantly less abundant in the ileum and cecum of healthy M5.1 chickens but become rapidly enriched following *Eimeria* infection. Additionally, *Weissella*, *S. gallinarum*, and *E. durans/hirae* are uniquely abundant in the ileum of healthy M5.1 chickens and are not diminished as much as in the other two breeds in response to *Eimeria* infection. These findings underscore the differential responses of the intestinal microbiome to coccidiosis among the three chicken breeds, highlighting the potential role of the intestinal microbiome in disease resistance. The commensal bacteria differentially present in Fayoumi chickens may represent promising probiotic candidates for mitigating coccidiosis and potentially other diseases.

## Supplementary Information

Additional file 1: Table S1. Statistical significance of weighted and unweighted UniFrac distances in the ileal microbiome of three chicken breeds in response to coccidiosis. Table S2. Relative abundancesand log_2_ fold changes of major ileal bacteria among three chicken breeds in response to coccidiosis. Table S3. Statistical significance of weighted and unweighted UniFrac distances in the cecal microbiome of three chicken breeds in response to coccidiosis. Table S4. Relative abundancesand log_2_ fold changes of major cecal bacteria among three chicken breeds in response to coccidiosis.

## Data Availability

Raw sequencing reads of this study were deposited in the NCBI GenBank SRA database under the accession number PRJNA1192005.

## Abbreviations

ASV: Amplicon sequence variant
dpi: Days post-infection
EM: *E. maxima*
OPG: Oocysts per gram of the feces
SCFA: Short-chain fatty acid
