# Supplementary material for: Breed-specific responses to coccidiosis in chickens: identification of intestinal bacteria linked to disease resistance
Source: J Anim Sci Biotechnol. 2025 May 8;16:65. doi: 10.1186/s40104-025-01202-z (PMC12060511; doi:10.1186/s40104-025-01202-z)
Supplement: Supplementary file 1 — Additional file 1: Table S1. Statistical significance of weighted and unweighted UniFrac distances in the ileal microbiome of three chicken breeds in response to coccidiosis. Table S2. Relative abundancesand log2 fold changes of major ileal bacteria among three chicken breeds in response to coccidiosis. Table S3. Statistical significance of weighted and unweighted UniFrac distances in the cecal microbiome of three chicken breeds in response to coccidiosis. Table S4. Relative abundancesand log2 fold changes of major cecal bacteria among three chicken breeds in response to coccidiosis. [file 40104_2025_1202_MOESM1_ESM.docx]

**Table S1.** Statistical significance of weighted and unweighted UniFrac distances in the ileal microbiome of three chicken breeds in response to coccidiosis

|  | Cobb-Mock | Cobb-EM | Ghs6-Mock | Ghs6-EM | M5.1-Mock | M5.1-EM |
| --- | --- | --- | --- | --- | --- | --- |
| Cobb-Mock |  | *R*^2^ = 0.338 *P* < 0.001 | *R*^2^ = 0.123 *P* = 0.007 | *R*^2^ = 0.354 *P* < 0.001 | *R*^2^ = 0.169 *P* < 0.001 | *R*^2^ = 0.287 *P* < 0.001 |
| Cobb-EM | *R*^2^ = 0.275 *P* < 0.001 |  | *R*^2^ = 0.356 *P* < 0.001 | *R*^2^ = 0.156 *P* < 0.001 | *R*^2^ = 0.357 *P* < 0.001 | *R*^2^ = 0.187 *P* < 0.001 |
| Ghs6-Mock | *R*^2^ = 0.202 *P* < 0.001 | *R*^2^ = 0.127 *P* = 0.002 |  | *R*^2^ = 0.359 *P* < 0.001 | *R*^2^ = 0.081 *P* = 0.040 | *R*^2^ = 0.265 *P* < 0.001 |
| Fhs6-EM | *R*^2^ = 0.489 *P* < 0.001 | *R*^2^ = 0.117 *P* = 0.008 | *R*^2^ = 0.219 *P* < 0.001 |  | *R*^2^ = 0.331 *P* < 0.001 | *R*^2^ = 0.126 *P* = 0.002 |
| M5.1-Mock | *R*^2^ = 0. 448 *P* < 0.001 | *R*^2^ = 0.287 *P* < 0.001 | *R*^2^ = 0.202 *P* < 0.001 | *R*^2^ = 0.534 *P* < 0.001 |  | *R*^2^ = 0.227 *P* < 0.001 |
| M5.1-EM | *R*^2^ = 0.388 *P* < 0.001 | *R*^2^ = 0.082 *P* = 0.094 | *R*^2^ = 0.114 *P* = 0.002 | *R*^2^ = 0.193 *P* < 0.001 | *R*^2^ = 0.328 P < 0.001 |  |

Note: Cobb, Leghorn Ghs6, and Fayoumi M5.1 chickens were challenged with 50,000 sporulated *E. maxima* (EM) or mock–infected on d 10. Ileal digesta samples (*n* = 10–15) were collected on d 17 and subjected to DNA isolation and 16S rRNA gene sequencing. The lower triangle (shaded in blue) represents weighted UniFrac distances between groups. The upper triangle (shaded in red) displays unweighted UniFrac distances. Statistical significance was determined using PERMANOVA with 999 permutations.

**Table S2.** Relative abundances (%) and Log_2_ fold changes (LFC) of major ileal bacteria among three chicken breeds in response to coccidiosis

|  | **Cobb** | | | **Ghs6** | | | **M5.1** | | |
| --- | --- | --- | --- | --- | --- | --- | --- | --- | --- |
|  | Mock | EM | LFC | Mock | EM | LFC | Mock | EM | LFC |
| **Phylum** |  |  |  |  |  |  |  |  |  |
| Bacillota | 76.58 | 97.23 | 0.25 | 94.22 | 99.68 | 2.76^***^ | 95.99 | 95.85 | 0.13 |
| Actinomycetota | 22.91 | 0.75 | –6.71^***^ | 3.02 | 0.01 | –5.73^***^ | 0.61 | 0.04 | –4.21^***^ |
| Pseudomonadota | 0.13 | 2.01 | 1.54 | 2.46 | 0.31 | 0.38 | 3.28 | 4.01 | 0.32 |
| Cyanobacteriota | 0.38 | 0.01 | –5.14^***^ | 0.30 | 0.00 | –0.17 | 0.13 | 0.08 | –0.69 |
| Total | 100.00 | 100.00 |  | 100.00 | 100.00 |  | 100.00 | 99.98 |  |
| **Family** | | | | | | | | | |
| Lactobacillaceae | 62.35 | 82.96 | 1.11 | 73.58 | 99.21 | 2.25^***^ | 63.67 | 87.09 | 0.81 |
| Staphylococcaceae | 3.34 | 0.07 | –4.86^***^ | 7.08 | 0.08 | –4.76^***^ | 16.97 | 1.96 | –4.39^***^ |
| Enterococcaceae | 2.67 | 1.29 | –2.54 | 5.16 | 0.32 | –2.57^***^ | 10.20 | 4.82 | –1.21 |
| Corynebacteriaceae | 16.64 | 0.65 | –5.84^***^ | 2.11 | 0.01 | –6.23^***^ | 0.31 | 0.02 | –3.82^***^ |
| Clostridiaceae | 0.01 | 12.78 | 5.14^*^ | 0.09 | 0.00 | –0.09 | 0.01 | 0.61 | 3.29^*^ |
| Enterobacteriaceae | 0.01 | 2.01 | 5.06^***^ | 2.39 | 0.31 | –0.07 | 3.25 | 3.98 | 0.87 |
| Lachnospiraceae | 0.55 | 0.01 | –3.88^***^ | 4.36 | 0.04 | –3.78^***^ | 1.46 | 1.11 | –0.79 |
| Mycobacteriaceae | 3.94 | 0.10 | –5.66^***^ | 0.15 | 0.00 | –3.38^***^ | 0.02 | 0.00 | –1.14 |
| Peptostreptococcaceae | 2.94 | 0.08 | –4.7^**^ | 0.67 | 0.00 | –4.33^***^ | 0.02 | 0.02 | –0.55 |
| Aerococcaceae | 3.53 | 0.01 | –7.01^***^ | 0.06 | 0.00 | –1.75^***^ | 0.03 | 0.00 | –1.54 |
| Total | 95.98 | 99.96 |  | 95.65 | 99.97 |  | 95.94 | 99.61 |  |
| **Genus** | | | | | | | | | |
| *Lactobacillus* | 41.93 | 62.69 | 1.00 | 41.96 | 76.80 | 2.16^*^ | 33.80 | 61.82 | 1.6 |
| *Ligilactobacillus* | 16.61 | 12.86 | –0.2 | 28.16 | 13.27 | –0.49 | 6.63 | 19.97 | 2.7^**^ |
| *Weissella* | 0.07 | 0.00 | –2.93^***^ | 3.06 | 0.03 | –6.06^***^ | 22.45 | 3.95 | –4.01^***^ |
| *Staphylococcus* | 1.08 | 0.04 | –4.37^***^ | 6.79 | 0.08 | –5.7^***^ | 16.93 | 1.96 | –4.4^***^ |
| *Enterococcus* | 2.67 | 1.29 | –2.97 | 5.16 | 0.32 | –3.53^***^ | 10.20 | 4.82 | –1.23 |
| *Limosilactobacillus* | 3.67 | 7.38 | 1.66 | 0.38 | 9.11 | 5.19^***^ | 0.56 | 1.23 | 1.53 |
| *Corynebacterium* | 20.58 | 0.75 | –6.28^***^ | 2.25 | 0.01 | –7.21^***^ | 0.32 | 0.02 | –3.84^***^ |
| *Clostridium* | 0.00 | 12.75 | 4.7 | 0.06 | 0.00 | –1.07 | 0.01 | 0.60 | 3.27 |
| *Escherichia* | 0.01 | 2.01 | 4.63^**^ | 2.21 | 0.30 | –0.89 | 3.14 | 3.41 | 0.78 |
| *Romboutsia* | 2.92 | 0.08 | –5.14^***^ | 0.67 | 0.00 | –5.29^***^ | 0.02 | 0.02 | –0.62 |
| *Mediterraneibacter* | 0.25 | 0.00 | –4.18^***^ | 1.22 | 0.02 | –3.74^***^ | 0.48 | 0.46 | –0.42 |
| *Blautia* | 0.13 | 0.00 | –3.52^***^ | 1.20 | 0.00 | –5.01^***^ | 0.56 | 0.30 | –1.17 |
| *Brachybacterium* | 1.71 | 0.00 | –7.42^***^ | 0.46 | 0.00 | –5.7^***^ | 0.20 | 0.00 | –3.79^***^ |
| *Aerococcus* | 2.42 | 0.01 | –7.26^***^ | 0.05 | 0.00 | –2.44^***^ | 0.03 | 0.00 | –1.56 |
| *Jeotgalicoccus* | 2.08 | 0.02 | –6.04^***^ | 0.19 | 0.00 | –4.36^***^ | 0.02 | 0.00 | –1.43^***^ |
| *Sellimonas* | 0.05 | 0.00 | –2.54^***^ | 0.86 | 0.01 | –4.27^***^ | 0.14 | 0.12 | –0.38 |
| *Klebsiella* | 0.00 | 0.00 | 0.09 | 0.18 | 0.01 | –1.44 | 0.11 | 0.57 | 0.81 |
| *Lederbergia* | 0.07 | 0.00 | –2.77^***^ | 0.07 | 0.00 | –3.29^***^ | 0.92 | 0.00 | –5.66^***^ |
| *Ruoffia* | 1.11 | 0.00 | –6.49^***^ | 0.01 | 0.00 | –0.53^***^ | 0.00 | 0.00 | –0.1^***^ |
| *Chroobacteria* | 0.38 | 0.01 | –4.75^***^ | 0.30 | 0.00 | –2.09 | 0.13 | 0.08 | –0.5 |
| *Eubacterium* | 0.01 | 0.00 | –0.63^***^ | 0.63 | 0.00 | –1.9 | 0.03 | 0.00 | –1.72 |
| *Oceanobacillus* | 0.17 | 0.01 | –3.36^***^ | 0.13 | 0.00 | –3.65^***^ | 0.45 | 0.01 | –4.47^***^ |
| *Faecalibacterium* | 0.03 | 0.00 | –1.3 | 0.53 | 0.00 | –1.44 | 0.02 | 0.00 | –1.31^***^ |
| *Bacillaceae_*Unclassified | 0.08 | 0.00 | –0.53^***^ | 0.08 | 0.00 | –1.08^***^ | 0.39 | 0.00 | –0.87^***^ |
| *Massilimicrobiota* | 0.00 | 0.00 | –0.39 | 0.27 | 0.00 | –3.36^***^ | 0.03 | 0.12 | –0.04 |
| Total | 98.03 | 99.90 |  | 96.88 | 99.96 |  | 97.57 | 99.46 |  |
| **ASV** | | | | | | | | | |
| *Lactobacillus*_A*_*F1 | 31.23 | 44.73 | 0.91 | 34.86 | 64.45 | 1.95 | 2.14 | 36.18 | 5.22^***^ |
| *Lactobacillus johnsonii_*F3 | 10.70 | 17.95 | 1.3 | 7.10 | 12.33 | 2.73 | 31.66 | 25.64 | 0.06 |
| *Ligilactobacillus salivarius_*F2 | 16.61 | 12.86 | –0.27 | 28.16 | 13.26 | –0.68 | 6.63 | 19.97 | 2.61^*^ |
| *Staphylococcus gallinarum_*F8 | 0.39 | 0.01 | –4.91^***^ | 6.79 | 0.08 | –6.12^***^ | 16.93 | 1.78 | –4.73^***^ |
| *Weissella paramesenteroides_*F11 | 0.05 | 0.00 | –2.97^***^ | 2.63 | 0.02 | –6.28^***^ | 18.41 | 3.02 | –4.3^***^ |
| *Clostridium perfringens_*F15 | 0.00 | 12.73 | 4.37 | 0.05 | 0.00 | –0.58^***^ | 0.01 | 0.56 | 2.8 |
| *Enterococcus durans/hirae_*F20 | 1.60 | 0.17 | –3.55 | 2.00 | 0.08 | –3.26^***^ | 8.43 | 1.46 | –3.49^***^ |
| *Limosilactobacillus reuteri_*F18 | 0.10 | 2.11 | 4.23 | 0.15 | 7.83 | 6.16^***^ | 0.00 | 0.63 | 4.21^***^ |
| *Escherichia_*F5 | 0.01 | 2.01 | 4.6^*^ | 2.21 | 0.30 | –1.07 | 3.14 | 3.41 | 0.69 |
| *Enterococcus cecorum_*F16 | 0.98 | 1.12 | –2.51 | 2.84 | 0.24 | –3.35^*^ | 0.37 | 3.35 | 2.27 |
| *Corynebacterium stationis_*F36 | 5.64 | 0.24 | –6.04^***^ | 0.95 | 0.00 | –6.61^***^ | 0.13 | 0.00 | –3.45^***^ |
| *Weissella confusa/cibaria_*F39 | 0.00 | 0.00 | –0.17^***^ | 0.35 | 0.00 | –4.56^***^ | 3.67 | 0.93 | –3.55^**^ |
| *Corynebacterium stationis_*F46 | 3.52 | 0.15 | –5.84^***^ | 0.43 | 0.00 | –5.42^***^ | 0.08 | 0.00 | –2.91^**^ |
| *Limosilactobacillus reuteri_*F43 | 1.52 | 1.52 | –1.73 | 0.09 | 0.09 | –0.91 | 0.26 | 0.22 | –1.6 |
| *Corynebacterium_*F48 | 3.94 | 0.10 | –6.15^***^ | 0.15 | 0.00 | –4.54^***^ | 0.02 | 0.00 | –1.24 |
| *Corynebacterium_*F49 | 3.82 | 0.11 | –6.52^***^ | 0.12 | 0.00 | –4.07^***^ | 0.02 | 0.00 | –1.4 |
| *Romboutsia timonensis_*F26 | 2.92 | 0.08 | –5.19^**^ | 0.67 | 0.00 | –5.48^***^ | 0.02 | 0.02 | –0.71 |
| *Limosilactobacillus reuteri_*F47 | 1.21 | 1.38 | –0.1 | 0.07 | 0.11 | 0.45 | 0.16 | 0.20 | –0.75 |
| *Corynebacterium casei_*F56 | 2.84 | 0.11 | –5.64^***^ | 0.43 | 0.00 | –5.83^***^ | 0.05 | 0.00 | –2.11 |
| *Limosilactobacillus pontis_*F45 | 0.02 | 1.42 | 5.51^**^ | 0.00 | 0.91 | 4.52^**^ | 0.00 | 0.00 | –0.07^***^ |
| *Brachybacterium_*F72 | 1.71 | 0.00 | –7.47^***^ | 0.46 | 0.00 | –5.89^***^ | 0.20 | 0.00 | –3.88^***^ |
| *Jeotgalicoccus aerolatus_*F75 | 2.08 | 0.02 | –6.12^***^ | 0.19 | 0.00 | –4.54^***^ | 0.02 | 0.00 | –1.51^***^ |
| *Limosilactobacillus reuteri_*F78 | 0.80 | 0.66 | –1.56 | 0.05 | 0.04 | –0.63 | 0.12 | 0.10 | –1.28 |
| *Aerococcus_*A*_*F92 | 1.54 | 0.00 | –6.88^***^ | 0.04 | 0.00 | –2.35^***^ | 0.03 | 0.00 | –1.41 |
| *Enterococcus_*F93 | 0.01 | 0.00 | –0.75 | 0.16 | 0.00 | –3.92^***^ | 1.09 | 0.01 | –6.78^***^ |
| *Blautia_*F4 | 0.03 | 0.00 | –2.54^***^ | 0.50 | 0.00 | –4.67^***^ | 0.33 | 0.22 | –0.89 |
| *Mediterraneibacter_*F10 | 0.01 | 0.00 | –0.89 | 0.51 | 0.01 | –2.89 | 0.36 | 0.00 | –4.23^***^ |
| *Ruoffia tabacinasalis_*F106 | 1.11 | 0.00 | –6.55^***^ | 0.01 | 0.00 | –0.72^***^ | 0.00 | 0.00 | –0.19^***^ |
| *Lederbergia galactosidilytica_*F111 | 0.04 | 0.00 | –2.27^*^ | 0.06 | 0.00 | –3.45^***^ | 0.87 | 0.00 | –5.74^***^ |
| *Chroobacteria_*F114 | 0.37 | 0.01 | –4.78^***^ | 0.30 | 0.00 | –2.25 | 0.13 | 0.08 | –0.53 |
| *Staphylococcus xylosus_*F115 | 0.66 | 0.03 | –4.31^***^ | 0.00 | 0.00 | 0.3 | 0.00 | 0.18 | 3.68^***^ |
| *Eubacterium_*F34 | 0.01 | 0.00 | –0.69^***^ | 0.63 | 0.00 | –2.09 | 0.03 | 0.00 | –1.79 |
| *Aerococcus_*A*_*F126 | 0.88 | 0.00 | –6.42^***^ | 0.01 | 0.00 | –0.81^***^ | 0.01 | 0.00 | –0.42^***^ |
| *Mediterraneibacter_*F22 | 0.07 | 0.00 | –2.6 | 0.20 | 0.01 | –3.16^**^ | 0.00 | 0.33 | 3.48^**^ |
| *Sellimonas intestinalis_*F12 | 0.03 | 0.00 | –2.06 | 0.50 | 0.01 | –4.03^***^ | 0.01 | 0.05 | 0.88 |
| *Corynebacterium stationis_*F131 | 0.68 | 0.03 | –4.73^***^ | 0.07 | 0.00 | –3.1^***^ | 0.01 | 0.00 | –0.94 |
| *Blautia obeum_*F28 | 0.06 | 0.00 | –3.06^***^ | 0.42 | 0.00 | –4.49^***^ | 0.05 | 0.02 | –1.75 |
| *Enterococcus_*F142 | 0.07 | 0.01 | –2.76 | 0.16 | 0.00 | –2.51 | 0.28 | 0.00 | –0.89^***^ |
| *Massilimicrobiota timonensis_*F9 | 0.00 | 0.00 | –0.46 | 0.27 | 0.00 | –3.55^***^ | 0.03 | 0.12 | –0.13 |
| *Klebsiella granulomatis_*F150 | 0.00 | 0.00 | 0.01 | 0.07 | 0.00 | –1.7 | 0.07 | 0.27 | 0.22 |
| *Weissella_*F151 | 0.01 | 0.00 | –1.11 | 0.07 | 0.01 | –0.76 | 0.37 | 0.00 | –1.86 |
| *Mediterraneibacter_*F7 | 0.08 | 0.00 | –2.78^*^ | 0.23 | 0.00 | –3.04^*^ | 0.03 | 0.05 | 0.45 |
| *Pediococcus pentosaceus_*F161 | 0.02 | 0.00 | –1.44 | 0.02 | 0.00 | –2.03^***^ | 0.26 | 0.08 | –3.04^**^ |
| *Sellimonas monacensis_*F25 | 0.01 | 0.00 | –1.26^***^ | 0.21 | 0.00 | –3.17^***^ | 0.07 | 0.04 | –0.82 |
| *Limosilactobacillus_*F144 | 0.00 | 0.13 | 2.27 | 0.02 | 0.10 | 2.21 | 0.00 | 0.06 | 2.06^***^ |
| *Faecalibacterium_*F6 | 0.02 | 0.00 | –1.02 | 0.28 | 0.00 | –1.1^***^ | 0.01 | 0.00 | –0.71^***^ |
| *Klebsiella_*F167 | 0.00 | 0.00 | 0.01 | 0.02 | 0.00 | –0.4 | 0.03 | 0.23 | 0.89 |
| *Anaerobutyricum_*F23 | 0.01 | 0.00 | –1.53^**^ | 0.16 | 0.00 | –3.35^***^ | 0.03 | 0.09 | 0.46 |
| *Oceanobacillus chironomi_*F168 | 0.04 | 0.00 | –2.31^*^ | 0.07 | 0.00 | –2.91^***^ | 0.22 | 0.00 | –3.23^***^ |
| *Brevibacterium senegalense_*F177 | 0.32 | 0.00 | –5.29^***^ | 0.03 | 0.00 | –1.21^***^ | 0.01 | 0.00 | –1.2^***^ |
| Total | 97.77 | 99.69 |  | 95.77 | 99.88 |  | 96.80 | 99.30 |  |

Note: Day–of–hatch Cobb, Leghorn Ghs6, and Fayoumi M5.1 chickens were challenged with 50,000 sporulated *E. maxima* (EM) or mock–infected on d 10. The ileal digesta samples (*n* = 10–15) were collected on d 17 and subjected to DNA isolation and 16S rRNA gene sequencing. The average relative abundances (%) of the top 4 phyla, top 10 families, top 15 genera, and top 50 amplicon sequence variants (ASVs) of the ileal microbiota are shown, and the log_2_ fold change (LFC) and statistical significance were determined using ANCOM–BC (30). **P* < 0.05, ***P* < 0.01, and ****P* < 0.001.

**Table S3.** Statistical significance of weighted and unweighted UniFrac distances in the cecal microbiome of three chicken breeds in response to coccidiosis

|  | Cobb–Mock | Cobb–EM | Ghs6–Mock | Ghs6–EM | M5.1–Mock | M5.1–EM |
| --- | --- | --- | --- | --- | --- | --- |
| Cobb–Mock |  | *R*^2^ = 0.143 *P* < 0.001 | *R*^2^ = 0.168 *P* < 0.001 | *R*^2^ = 0.187 *P* < 0.001 | *R*^2^ = 0.211 *P* < 0.001 | *R*^2^ = 0.402 *P* < 0.001 |
| Cobb–EM | *R*^2^ = 0.321 *P* < 0.001 |  | *R*^2^ = 0.170 *P* < 0.001 | *R*^2^ = 0.116 *P* < 0.001 | *R*^2^ = 0.168 *P* < 0.001 | *R*^2^ = 0.290 *P* < 0.001 |
| Ghs6–Mock | *R*^2^ = 0.148 *P* = 0.028 | *R*^2^ = 0.323 *P* < 0.001 |  | *R*^2^ = 0.152 *P* < 0.001 | *R*^2^ = 0.110 *P* < 0.001 | *R*^2^ = 0.359 *P* < 0.001 |
| Ghs6–EM | *R*^2^ = 0.421 *P* < 0.001 | *R*^2^ = 0.108 *P* = 0.013 | *R*^2^ = 0.369 *P* < 0.001 |  | *R*^2^ = 0.151 *P* < 0.001 | *R*^2^ = 0.299 *P* < 0.001 |
| M5.1–Mock | *R*^2^ = 0.192 *P* = 0.006 | *R*^2^ = 0.249 *P* = 0.002 | *R*^2^ = 0.068 *P* = 0.134 | *R*^2^ = 0.321 *P* < 0.001 |  | *R*^2^ = 0.308 *P* < 0.001 |
| M5.1–EM | *R*^2^ = 0.408 *P* < 0.001 | *R*^2^ = 0.109 *P* = 0.040 | *R*^2^ = 0.361 *P* < 0.001 | *R*^2^ = 0.075 *P* = 0.100 | R^2^ = 0.312 *P* < 0.001 |  |

Note: Cobb, Leghorn Ghs6, and Fayoumi M5.1 chickens were challenged with 50,000 sporulated *E. maxima* (EM) or mock–infected on d 10. Cecal digesta samples (*n* = 10–15) were collected on d 17 and subjected to DNA isolation and 16S rRNA gene sequencing. The lower triangle (shaded in blue) represents weighted UniFrac distances between groups. The upper triangle (shaded in red) displays unweighted UniFrac distances. Statistical significance was determined using PERMANOVA with 999 permutations.

**Table S4.** Relative abundances (%) and Log_2_ fold changes (LFC) of major cecal bacteria among three chicken breeds in response to coccidiosis

|  | **Cobb** | | | **Ghs6** | | | **M5.1** | | |
| --- | --- | --- | --- | --- | --- | --- | --- | --- | --- |
|  | Mock | EM | LFC | Mock | EM | LFC | Mock | EM | LFC |
| **Phylum** | | | | | | | | | |
| Bacillota | 99.75 | 93.07 | 0.04 | 98.76 | 98.74 | 0.14 | 95.32 | 95.49 | 0.17 |
| Pseudomonadota | 0.13 | 6.06 | 4.69^***^ | 1.06 | 0.77 | –0.33 | 4.20 | 4.33 | 0.04 |
| Actinomycetota | 0.12 | 0.86 | 2.64^***^ | 0.18 | 0.49 | 1.36^***^ | 0.47 | 0.19 | –1.56^**^ |
| Mycoplasmatota | 0.00 | 0.00 | –0.01 | 0.00 | 0.00 | –0.61 | 0.01 | 0.00 | –1.43^***^ |
| Total | 100.00 | 99.99 |  | 100.00 | 100.00 |  | 100.00 | 100.00 |  |
| **Family** | | | | | | | | | |
| Lachnospiraceae | 48.43 | 52.50 | 0.29 | 52.79 | 52.09 | 0.14 | 52.45 | 49.99 | –0.32 |
| Oscillospiraceae | 30.13 | 12.39 | –1.13^***^ | 27.06 | 11.71 | –1.17^***^ | 26.36 | 6.82 | –2.06^***^ |
| Lactobacillaceae | 7.58 | 18.95 | 1.36 | 5.23 | 26.38 | 2.52^***^ | 1.60 | 23.00 | 4.36^***^ |
| Coprobacillaceae | 2.68 | 1.80 | –0.89 | 10.15 | 7.10 | –0.53 | 8.73 | 12.89 | 0.33 |
| Enterobacteriaceae | 0.13 | 5.82 | 4.63^***^ | 1.03 | 0.76 | –0.35 | 4.20 | 4.33 | 0.12 |
| Peptostreptococcaceae | 8.32 | 0.08 | –7.21^***^ | 0.18 | 0.00 | –5.24^***^ | 0.10 | 0.00 | –2.54^*^ |
| Enterococcaceae | 0.05 | 2.19 | 3.26 | 0.13 | 0.07 | –0.76 | 1.25 | 1.70 | 0.58 |
| Bacillaceae | 0.58 | 1.07 | –2.34 | 1.15 | 0.05 | –3.39 | 2.29 | 0.00 | –6.54^***^ |
| Clostridiaceae | 0.28 | 3.06 | 2.45 | 0.26 | 0.13 | –0.26 | 0.29 | 0.76 | 0.79 |
| Erysipelotrichaceae | 0.88 | 0.34 | –1.51 | 1.12 | 0.62 | –0.94 | 0.62 | 0.26 | –2.65 |
| Total | 99.06 | 98.20 |  | 99.10 | 98.91 |  | 97.89 | 99.75 |  |
| **Genus** | | | | | | | | | |
| *Mediterraneibacter* | 17.14 | 14.73 | –0.03 | 14.77 | 14.97 | 0.35 | 18.32 | 17.57 | –0.26 |
| *Blautia* | 8.72 | 8.48 | –0.01 | 13.08 | 11.23 | –0.01 | 11.01 | 8.60 | –0.69 |
| *Lactobacillus* | 3.30 | 8.04 | 1.5 | 2.59 | 19.30 | 3.65^***^ | 0.40 | 12.75 | 5.92^***^ |
| *Faecalibacterium* | 16.54 | 2.68 | –2.24 | 12.79 | 3.83 | –1.77 | 10.90 | 2.08 | –4.47^*^ |
| *Sellimonas* | 4.55 | 8.37 | 0.82 | 7.11 | 8.79 | 0.4 | 4.13 | 4.60 | –0.22 |
| *Ligilactobacillus* | 3.65 | 9.39 | 1.11 | 2.58 | 5.86 | 1.13 | 1.14 | 9.14 | 3.39^***^ |
| *Lachnospiraceae*_Unclassified | 6.53 | 5.23 | –0.58 | 5.94 | 4.95 | –0.13 | 4.64 | 2.21 | –1.28 |
| *Anaerostipes* | 1.60 | 7.44 | 1.51 | 1.04 | 4.78 | 2.38^***^ | 1.71 | 8.18 | 2.68 |
| *Massilimicrobiota* | 1.20 | 0.50 | –1.54 | 5.33 | 2.90 | –1.47 | 4.25 | 7.08 | 0.49 |
| *Escherichia* | 0.13 | 5.82 | 4.65^***^ | 1.03 | 0.76 | –0.35 | 4.19 | 4.32 | 0.13 |
| *Gemmiger* | 5.40 | 1.01 | –2.87^**^ | 4.96 | 1.76 | –1.92 | 0.97 | 1.18 | –0.09 |
| *Thomasclavelia* | 0.55 | 0.64 | –0.3 | 2.86 | 2.23 | –0.53 | 3.35 | 4.26 | 0.25 |
| *Eisenbergiella* | 0.91 | 1.31 | 0.71 | 2.91 | 1.39 | –1.04 | 2.94 | 1.75 | –1.86 |
| *Enterocloster* | 2.21 | 1.21 | –1.31 | 2.38 | 0.42 | –2.86^***^ | 2.38 | 2.73 | –0.22 |
| *Anaerobutyricum* | 0.53 | 2.69 | 2.29 | 0.90 | 3.16 | 1.88^***^ | 0.54 | 2.45 | 1.88 |
| *Oscillospiraceae*_Unclassified | 2.44 | 1.37 | –0.94 | 1.75 | 1.09 | –0.4 | 2.14 | 0.67 | –1.83^***^ |
| *Coprobacillaceae*_Unclassified | 0.94 | 0.66 | –1.28 | 1.96 | 1.97 | 0.1 | 1.13 | 1.56 | –0.2 |
| *Romboutsia* | 8.29 | 0.08 | –7.2^***^ | 0.16 | 0.00 | –4.86^***^ | 0.01 | 0.00 | –1.18 |
| *Eubacterium* | 1.04 | 0.57 | –1.76 | 1.47 | 0.92 | –0.53 | 2.43 | 0.31 | –5.16^***^ |
| *Negativibacillus* | 0.86 | 0.79 | –0.58 | 1.33 | 1.28 | –0.07 | 1.40 | 0.60 | –0.32 |
| *Murimonas* | 0.58 | 0.18 | –1.44 | 1.42 | 0.74 | –1.33 | 2.34 | 0.76 | –0.97 |
| *Enterococcus* | 0.05 | 2.19 | 3.27 | 0.13 | 0.07 | –0.76 | 1.25 | 1.70 | 0.59 |
| *Bacillaceae*_Unclassified | 0.58 | 1.07 | –2.34 | 1.15 | 0.05 | –3.48 | 2.28 | 0.00 | –5.8^***^ |
| *Clostridium* | 0.28 | 3.06 | 2.47 | 0.26 | 0.13 | –0.26 | 0.29 | 0.76 | 0.81 |
| *Pseudoflavonifractor* | 0.18 | 0.72 | 1.77^*^ | 0.44 | 0.30 | 0.04 | 3.04 | 0.20 | –2.57^***^ |
| Total | 88.20 | 88.23 |  | 90.34 | 92.88 |  | 87.18 | 95.46 |  |
| **ASV** | | | | | | | | | |
| *Lactobacillus*_A*_*F1 | 2.89 | 6.73 | 1.46 | 2.30 | 18.75 | 3.68^***^ | 0.10 | 10.41 | 7.57^***^ |
| *Ligilactobacillus salivarius_*F2 | 3.65 | 9.38 | 1.1 | 2.58 | 5.85 | 1.13 | 1.14 | 9.14 | 3.39^***^ |
| *Blautia_*F4 | 2.20 | 2.17 | –1.0 | 6.87 | 5.97 | –0.14 | 5.02 | 4.55 | –1.2 |
| *Faecalibacterium_*F6 | 8.64 | 1.42 | –2.39 | 7.15 | 2.06 | –1.85 | 6.26 | 1.20 | –4.79^*^ |
| *Mediterraneibacter_*F7 | 6.99 | 2.49 | –3.2 | 3.85 | 2.10 | –0.61 | 4.27 | 6.54 | –0.72 |
| *Massilimicrobiota timonensis_*F9 | 1.20 | 0.50 | –1.57 | 5.33 | 2.90 | –1.47 | 4.25 | 7.08 | 0.49 |
| *Mediterraneibacter_*F10 | 0.83 | 1.77 | 1.57 | 5.21 | 6.34 | 1.2 | 5.64 | 0.67 | –6.42^*^ |
| *Sellimonas intestinalis_*F12 | 2.80 | 5.23 | –0.38 | 4.98 | 5.11 | –0.82 | 0.53 | 1.54 | 0.12 |
| *Faecalibacterium_*F13 | 6.55 | 0.96 | –2.15 | 4.55 | 1.44 | –1.64 | 3.65 | 0.64 | –3.78 |
| *Anaerostipes butyraticus_*F14 | 0.70 | 3.98 | 1.46 | 0.40 | 3.49 | 3.1^***^ | 0.23 | 6.98 | 4.73^***^ |
| *Escherichia_*F5 | 0.09 | 5.80 | 5.02^***^ | 0.97 | 0.76 | –0.32 | 4.09 | 4.32 | 0.14 |
| *Gemmiger gallinarum_*F17 | 5.40 | 1.01 | –2.89^*^ | 4.96 | 1.76 | –1.93 | 0.97 | 1.18 | –0.1 |
| *Thomasclavelia spiroformis_*F19 | 0.55 | 0.64 | –0.33 | 2.85 | 2.22 | –0.53 | 2.80 | 4.15 | 0.1 |
| *Lachnospiraceae_*F21 | 1.04 | 2.30 | 0.29 | 3.08 | 3.57 | 0.26 | 1.31 | 0.40 | –3.9 |
| *Mediterraneibacter_*F22 | 4.15 | 1.95 | –1.23 | 0.00 | 3.08 | 4.95^***^ | 0.00 | 2.73 | 3.85^***^ |
| *Anaerobutyricum_*F23 | 0.53 | 2.69 | 2.27 | 0.90 | 3.16 | 1.88^***^ | 0.54 | 2.45 | 1.88 |
| *Mediterraneibacter_*F24 | 1.29 | 1.24 | 0.19 | 3.30 | 1.38 | –4.85 | 3.14 | 0.32 | –4.85 |
| *Sellimonas monacensis_*F25 | 1.06 | 1.86 | 1 | 1.21 | 2.12 | 0.95^**^ | 1.64 | 1.64 | –0.38 |
| *Eisenbergiella_*F27 | 0.88 | 1.02 | –0.56 | 2.38 | 1.00 | –2.48 | 2.51 | 1.69 | –1.59 |
| *Anaerostipes_*F29 | 0.91 | 3.29 | 0.69 | 0.64 | 1.29 | 1.05 | 1.25 | 1.20 | –1 |
| *Blautia obeum_*F28 | 1.87 | 1.44 | –0.26 | 2.85 | 0.85 | –1.75 | 1.36 | 0.20 | –4.8^**^ |
| *Blautia_*F30 | 1.52 | 2.93 | 1.73 | 0.49 | 1.45 | 2.02 | 0.75 | 0.77 | –0.95 |
| *Blautia_*F31 | 2.08 | 0.54 | –1.77 | 1.22 | 1.49 | 0.66 | 1.98 | 0.45 | –2.65 |
| *Romboutsia timonensis_*F26 | 8.29 | 0.08 | –7.21^***^ | 0.16 | 0.00 | –4.86^***^ | 0.01 | 0.00 | –1.18 |
| *Murimonas intestini_*F32 | 0.50 | 0.17 | –1.41 | 1.41 | 0.74 | –1.33 | 2.34 | 0.76 | –0.97 |
| *Coprobacillaceae_*F33 | 0.43 | 0.50 | –0.45 | 1.12 | 1.47 | 0.55 | 0.60 | 1.48 | 0.68 |
| *Sellimonas intestinalis_*F35 | 0.32 | 0.72 | 1.46 | 0.37 | 0.96 | 1.89^**^ | 1.91 | 1.23 | –1.11 |
| *Blautia_*F37 | 0.37 | 0.82 | 1.79 | 0.79 | 0.76 | 0.06 | 0.97 | 1.66 | –0.72 |
| *Lactobacillus johnsonii_*F3 | 0.41 | 1.30 | 1.66 | 0.29 | 0.55 | 4.13^*^ | 0.31 | 2.34 | 4.04^***^ |
| *Eubacterium_*F34 | 1.02 | 0.25 | –3.33^***^ | 1.18 | 0.80 | –0.32 | 1.68 | 0.20 | –5.68^***^ |
| *Enterococcus cecorum_*F16 | 0.02 | 2.16 | 3.53 | 0.08 | 0.05 | –0.48 | 1.12 | 1.60 | 0.81 |
| *Bacillaceae_*F38 | 0.58 | 1.07 | –2.35 | 1.15 | 0.05 | –3.48 | 2.28 | 0.00 | –5.81^***^ |
| *Negativibacillus massiliensis_*F40 | 0.56 | 0.57 | –0.79 | 0.95 | 0.86 | –0.12 | 0.96 | 0.40 | –0.78 |
| *Enterocloster_*F41 | 1.07 | 0.39 | –2.34 | 0.94 | 0.06 | –5.63^***^ | 1.39 | 0.70 | –3.56 |
| *Mediterraneibacter hominis_*F42 | 0.22 | 0.34 | 1.44 | 0.15 | 0.44 | 1.5 | 1.14 | 1.71 | –0.19 |
| *Faecalibacterium_*F44 | 1.06 | 0.19 | –2.6 | 0.98 | 0.26 | –2.44 | 0.86 | 0.19 | –3.19 |
| *Mediterraneibacter_*F50 | 0.41 | 1.18 | –0.45 | 0.26 | 0.02 | –2.6 | 0.51 | 0.77 | 1.44 |
| *Clostridium perfringens_*F15 | 0.00 | 2.88 | 4.31^***^ | 0.00 | 0.00 | 0 | 0.01 | 0.14 | 1.83 |
| *Mediterraneibacter_*F52 | 0.27 | 2.18 | 1.9 | 0.14 | 0.33 | 1.66 | 0.01 | 0.00 | 0.07 |
| *Blautia faecicola_*F51 | 1.10 | 0.11 | –2.16 | 0.61 | 0.10 | –1.83 | 0.82 | 0.40 | –3.87 |
| *Enterocloster_*F53 | 0.37 | 0.48 | 0.5 | 0.30 | 0.17 | –0.29 | 0.12 | 1.46 | 4.6 |
| *Anaerotruncus colihominis_*F55 | 0.35 | 0.79 | 0.14 | 0.72 | 0.23 | –1.31 | 0.54 | 0.13 | –3.51^*^ |
| *Acutalibacter_*F54 | 0.33 | 0.40 | –0.33 | 0.49 | 0.42 | –0.19 | 0.80 | 0.29 | –2.81 |
| *Erysipelotrichaceae_*F57 | 0.72 | 0.23 | –2.41 | 0.81 | 0.40 | –1.34 | 0.49 | 0.14 | –4.03 |
| *Merdimonas faecis_*F59 | 0.39 | 1.12 | 1.17 | 0.36 | 0.19 | –1.34 | 0.63 | 0.01 | –5.76^***^ |
| *Blautia_*F60 | 0.45 | 0.39 | 0.27 | 0.51 | 0.52 | –0.48 | 0.54 | 0.22 | –3.2 |
| *Coprobacillaceae_*F58 | 0.50 | 0.16 | –3.53^**^ | 0.84 | 0.50 | –0.74 | 0.53 | 0.08 | –4.63^**^ |
| *Ruthenibacterium_*F61 | 0.11 | 1.39 | 2.52 | 0.24 | 0.26 | 0.16 | 0.36 | 0.05 | –3.94^***^ |
| *Sellimonas intestinalis_*F62 | 0.37 | 0.56 | –1.02 | 0.55 | 0.60 | 0.25 | 0.05 | 0.20 | 1.41 |
| *Limosilactobacillus reuteri_*F18 | 0.12 | 0.73 | 2.84 | 0.00 | 0.70 | 6.06^***^ | 0.00 | 0.71 | 5.7^***^ |
| Total | 78.16 | 82.50 |  | 83.47 | 89.58 |  | 74.41 | 87.12 |  |

Note: Cobb, Leghorn Ghs6, and Fayoumi M5.1 chickens were challenged with 50,000 sporulated *E. maxima* (EM) or mock–infected on d 10. Cecal digesta samples (*n* = 10–15) were collected on d 17 and subjected to DNA isolation and 16S rRNA gene sequencing. The average relative abundances (%) of the top 4 phyla, top 10 families, top 15 genera, and top 50 amplicon sequence variants (ASVs) of the cecal microbiota are shown, and log_2_ fold changes (LFC) and statistical significance were determined using ANCOM–BC (30). **P* < 0.05, ***P* < 0.01, and ****P* < 0.001.
